# Emergence of Scale-Free Leadership Structure in Social Recommender Systems

**DOI:** 10.1371/journal.pone.0020648

**Published:** 2011-07-27

**Authors:** Tao Zhou, Matúš Medo, Giulio Cimini, Zi-Ke Zhang, Yi-Cheng Zhang

**Affiliations:** 1 Web Sciences Center, University of Electronic Science and Technology of China, Chengdu, People's Republic of China; 2 Department of Physics, University of Fribourg, Fribourg, Switzerland; 3 Department of Modern Physics, University of Science and Technology of China, Hefei, People's Republic of China; University of Maribor, Slovenia

## Abstract

The study of the organization of social networks is important for the understanding of opinion formation, rumor spreading, and the emergence of trends and fashion. This paper reports empirical analysis of networks extracted from four leading sites with social functionality (Delicious, Flickr, Twitter and YouTube) and shows that they all display a scale-free leadership structure. To reproduce this feature, we propose an adaptive network model driven by social recommending. Artificial agent-based simulations of this model highlight a “good get richer” mechanism where users with broad interests and good judgments are likely to become popular leaders for the others. Simulations also indicate that the studied social recommendation mechanism can gradually improve the user experience by adapting to tastes of its users. Finally we outline implications for real online resource-sharing systems.

## Introduction

Social network analysis has become a joint focus of many branches of science [1], [2]. Various social networks have been systematically investigated, such as friendship, membership and co-authorship networks. In this work we focus on the so-called leadership networks which capture how people copy actions or receive information from others. Although they play a significant role in formation and propagation of social opinions, leadership networks have received considerably less attention than other social networks–possibly because of the lack of empirical data. Recently, some researchers reported the emergence of scale-free leadership structures from initially homogeneous interaction networks in evolutionary games, such as the *minority game* [3], [4], [5], the *ultimatum game* [6] and the prisoner's dilemma game [7], [8], [9], [10], where agent 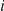 is considered to be led by agent 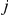 if 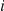 has adopted 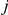
's strategy. Since it is hard to automatically extract *who follows whom* from records of economic activities, up to now no empirical evidence has been reported to either support or challenge these findings for economic systems. On the other hand, web activity data give us the possibility to study leadership structures in the process of information propagation. In this paper, we report both empirical evidence and a theoretical model for the emergence of scale-free leadership networks in online societies. Furthermore, we discuss which user characteristics are important for becoming a leader.

Beyond providing a mechanism leading to scale-free leadership structures, this work can contribute to solving the information overload problem created by the unceasingly growing amount of easily available information. *Recommender systems* provide a solution to this problem by analyzing users' profiles and past preferences and using them for automated recommendation of relevant items to individual users [11]. The majority of current recommender systems use a centralized approach where all data is stored and analyzed at one place. Typical algorithms include collaborative filtering [12], [13], matrix decomposition [14], [15], [16], and spreading processes [17], [18], [19]. However, this paradigm is challenged by the findings that social influence often plays a more important role than similarity of past activities [20], [21] and recommendations made by a system are preferred less than those coming from our friends [22], [23]. In response, *social recommendation* has become a candidate for the next recommendation paradigm [24]. *Social recommender systems* can be designed (i) in a passive way where a user selects other users as information sources and can import URLs or subscribe blog articles from them (as in *delicious.com* and *blogger.com*) [25] or (ii) in an active way where each user can recommend items to other users who have accepted him as information source (as in *douban.com* and *twitter.com*) [26]. While very different from the user's point of view, these two ways are similar in how information favored by one user spreads to the user's followers, followers' followers, and so on [27], [28], [29]. This process is similar to the well-studied epidemic spreading on networks [30], [31]. The model proposed and investigated here mimics information spreading process in adaptive social networks. We evaluate its efficiency in filtering out the low-quality and irrelevant information and show that this distributed social recommender model can enhance the user experience.

## Results

### Empirical Results

The studied bookmarking data was obtained by crawling the publicly-available data from the social bookmarking website *delicious.com* [32]. The resulting network consists of 392 251 users and 1 686 131 directed links. We say that user 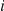 is a follower of user 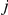 (or, equivalently, 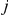 is a leader of 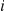) if 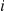 has imported some of 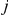
's bookmarks. In this way, a directed social network of users is constructed where each link represents a leader-follower relationship. We define the direction of each link as 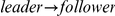 and thus the out-degree of a user (i.e., the number of user's followers) can be used to quantify the person's leadership strength. To obtain a solid understanding of the leadership structure, we study data from three other social sites containing this kind of structure: *flickr.com*, *twitter.com* and *youtube.com*. These data sets were provided upon request by [36] for *flickr.com* and *youtube.com* and by [37] for *twitter.com*. In the first two cases, user 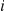 follows user 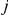 if 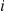 has asked user 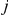 for friendship and user 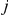 accepted this invitation. In the case of Twitter data, users can explicitly follow other users, who will in turn push messages to them.

**Figure 1:**
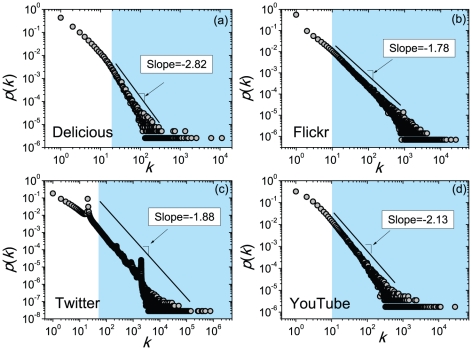
Scale-free leadership structure – empirical results. Out-degree distributions of the studied leadership networks and their power-law fits. Shaded areas in the figures show the range where the data is best described by a power-law distribution (they are delimited by 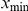 minimizing the 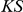 statistic).

Table 1 summarizes basic statistics of the studied leadership networks and results of power-law fits of their out-degree distributions based on the standard maximum likelihood estimation [33], [34]. The out-degree distributions themselves are shown in Fig. 1 together with their power-law fits in the range 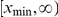 (according to [34], the optimal value of 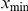 is the one yielding the minimal value of the Kolmogorov-Smirnov statistic).

**Table 1:** Basic characteristics and results of statistical analysis for the studied leadership networks.

Dataset	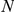	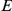	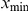	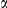	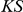
Delicious	392,251	1,686,131	20	2.82	0.010
Flickr	1,441,432	22,613,981	10	1.78	0.021
Twitter	35,689,148	1,468,365,183	50	1.88	0.033
YouTbue	570,774	4,945,382	10	2.13	0.013

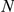
 represents the number of users, 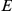 represents the number of links, 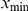 is the lower bound of the range fit by a power-law distribution, 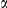 is the corresponding power-law exponent obtained by maximum likelihood estimation and 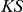 is the goodness-of-fit value based on the Kolmogorov-Smirnov statistic [34].

### Model

The modeled system consists of 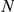 users, each having 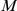 information sources (i.e., 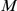 leaders). Nodes of the corresponding directed network are hence of identical in-degree 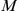. The out-degree can be used to quantify the node's leadership status (see also more complicated measures based on PageRank [35], [38] or LeaderRank [39] algorithms). At each time step, a randomly selected user posts an item (this generic term stands for an URL, a news, a blog article, a picture, a video, or any other shared content). This item is automatically considered to be approved (liked) by this user and spreads to all user's followers who consequently judge this item. If a follower approves the item, it spreads farther to the follower's followers. If the item is disapproved, it does not spread further from this disapproving node (though, it may continue to spread from some other nodes which approve it). Note that, in each time step, one piece of news is introduced and spreads through the whole system depending on approvals/disapprovals of users. This “fast user evaluations” mechanism simplifies implementation of the model and, according to our tests, has little impact on the essential features of the system's dynamics.

In the model, leaders are evaluated by their followers on the basis of how the followers appreciate recommendations coming from them. In particular, the similarity of evaluations 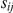 is computed for each leader-follower pair. If user 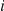 receives an item from user 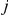 and approves it, the similarity score is updated as 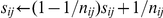 while when this item is disapproved by user 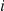, 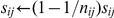. Here 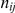 denotes the cumulative number of items that 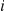 has received from 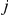. This form ensures that contribution of one incoming item to the similarity value is inversely proportional to the total number of items transferred through the corresponding channel. Each user is initially given 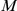 randomly selected leaders whose similarity values are set to 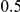. It is easy to prove that the aforementioned formulas lead to 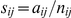 where 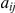 denotes the number of items received from 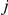 and approved by 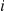.

To allow for a gradual evolution of the leader-follower network, each user updates their leaders after every 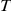 evaluated items. We adopt a simple approach in which the worst-performing leader (the one with the lowest similarity value) of user 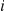 is dropped and replaced by a randomly selected user 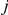 (given 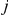 is not among the given user's leaders yet). Similarity of this new leader is set to 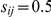 and the number of transferred items to 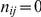, independently of whether 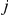 has been 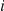
's leader sometimes before or not. Note that this updating is very economic as it requires no computation and no centralized data storage (compared with the expensive network optimization techniques studied in [27], [28]). Yet it ensures that the system evolves in a self-organized way and gradually adapts to the tastes of its users.

To test the described recommendation algorithm, we introduce a simple agent-based model. The cornerstone of this model is how to cast evaluations of items by users. We adopt the approach similar to [27] where users and items are described by 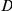-dimensional taste and attribute vectors, respectively. While elements of the user taste vectors 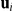 are randomly set to either 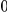 or 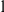 with equal probabilities, elements of the item attribute vectors 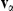 are independently drawn from the uniform distribution 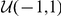. Note that for clarity we use Latin and Greek letters for user- and item-related indices, respectively. Opinion of user 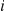 about item 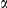 is modeled as 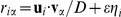 where 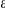 is a random variable drawn from the uniform distribution 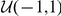 and 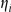 represents the evaluation noise magnitude of user 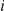 (the lower the 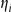, the better the judgment, and vice versa). In this way, opinion of a user about an item is of a high value if this user's taste vector highly overlaps with the news's attribute vector. Values 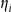 are drawn from the uniform distribution 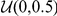 and stay fixed during the simulation. If 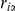 is larger than a certain threshold 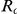, user 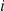 approves item 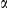. At every time step, after user 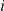 has been randomly selected to post item 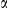, items with random attributes are generated until one is approved by this user (i.e., it satisfies the approval condition 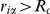). Spreading of this item then starts by pushing it to all followers of user 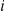.

This agent-based vector model has a simple intuitive interpretation. Respective item's attributes, ranging from 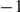 to 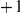, represent item's quality in various aspects (the higher, the better) as well as item's topic (e.g., if it concerns sport or politics or something else). Respective user's tastes, ranging from 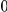 to 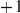, represent user's sensitivity to different item attributes. A user whose taste vector mostly consists of ones is sensitive to all attributes and hence can judge items well. By contrast, a user whose taste vector mostly consists of zeros is ignorant to most aspects and can be satisfied with items that would be judged badly by most users.

### Scale-Free Leadership Structure

**Figure 2:**
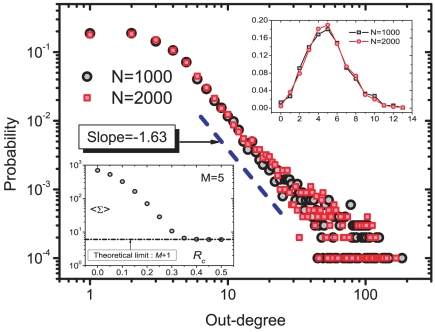
Scale-free leadership structure – simulation results. Out-degree distributions of the resulting leadership networks at time step 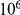 for 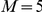, 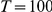, 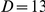, 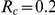 and different values of 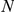. The upper-right inset displays the initial out-degree distributions. The lower-left inset shows the average number of readers of an item as a function of 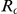 for 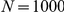. The thick dashed line with slope 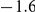 is shown as a guide to the eye. All data points reported here and later are averaged over 10 realizations.

The threshold 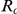 determines the average spreading range of items (i.e., their average number of readers 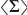). Although the approval thresholds could differ from one user to another, for simplicity we set them all identical. As shown in the lower-left inset of Fig. 2, 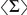 decreases quickly as 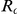 grows and approaches its lower bound when 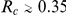 (each item is evaluated at least by the user who submitted it and all followers of this user, hence this lower bound equals 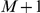). We set 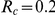 to achieve 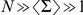. The upper-right inset of Fig. 2 shows the initial out-degree distributions which are naturally simple Poisson distributions peaked at 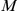. After a certain period of the system's evolution (Fig. 2 displays the results after 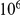 time steps), a scale-free leadership structure is created with the scaling exponent 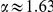.

**Figure 3:**
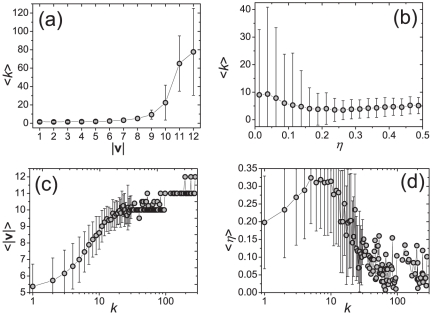
Broad interests and good judgments make a leader. Dependencies between the leadership strength and the scope of interests (a,c), and the quality of evaluations (b,d), respectively. The data points and error bars correspond to mean values and standard deviations. In (c) and (d), when 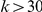, there is not enough data to obtain credible error bars, hence they are not shown. The population size is 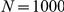; other parameter values are the same as in Fig. 2.

Scale-free networks are observed in very diverse systems [40] which indicates the existence of distinct mechanisms of their emergence [41]. While the majority of evolving network models are directly or implicitly inspired by the “rich get richer” phenomenon [42], [43], [44], there are plenty of other possible mechanisms such as the optimal design [45], Hamiltonian dynamics [46], merging and regeneration [47] and stability constraints [48]. The mechanism leading to scale-free structures in our model is different as it is based on a spreading mechanism in a social network and user heterogeneity. To uncover which factors make a popular leader, we characterize user 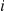 by the quality of evaluations and the scope of interests. The former is measured by the noise level 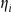 and the latter by the coverage 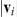 which we define as the sum of the taste vector's elements (which in our case is equal to the number of ones in 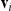). In Fig. 3, we report how the scope of interests and quality of evaluations affect the number of followers. As explained before, users with high 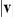 can better reveal intrinsic quality of items and hence they are likely to approve items with many positive entries in their attribute vectors–they are good filters of the content. If a user cannot find enough taste-mates (users with similar taste vectors), users who filter well can be used instead. Therefore, in accordance with the dependencies shown in Figs. 3a and 3c, users with high coverage usually have large numbers of followers. The role of quality of evaluations is more complicated. As shown in Fig. 3d, it is clear that popular leaders have small 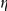. However, an accurate user may have a low popularity (see Fig. 3b: the average out-degree of accurate users is only slightly higher than that of inaccurate users) because however accurate user 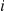 is, if his scope is not broad enough, the number of users with similar taste is limited.

We also studied the case where some users are more active than the others (they post and evaluate items more frequently). In the early stage, the active users have good chance to become popular leaders but in the long term, the popularity difference between active and normal users vanishes. This suggests that it is indeed the intrinsic personal profile–scope of interests and quality of evaluations–what plays the crucial role in determining a user's position in the social leadership network. We further investigated cases where (i) users have identical noise levels, (ii) users have identical coverage, (iii) users are all alike. In all these cases, the resulting out-degree distributions are considerably narrower than those reported in Fig. 2. Together with big standard deviations observed in Figs. 3a and 3b for large 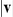 and small 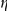, we can conclude that each of the qualities alone is not enough: popular leaders are those who have both broad scope and little randomness in their evaluations. This is similar to the “good get richer” mechanism proposed in the study of complex networks [49], [50].

### Numerical Validation of Social Recommending

**Figure 4:**
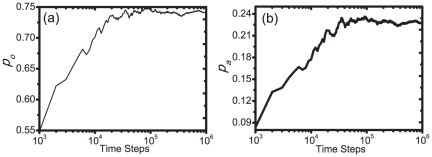
User experience is enhanced by the social recommender system. Probabilities of saying *ok* (a) and approving (b) items versus time. Values shown at time 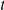 correspond to the average 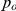 and 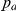 in time steps from 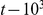 to 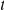. Parameter values are the same as in Fig. 3.

To verify whether the proposed social recommending mechanism and the network updating process can enhance the user experience, we study how users' responses to the recommended items change over time. In addition to user approval, we introduce a lower level of user satisfaction by assuming that user 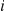 says *ok* to item 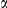 if 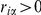. The ratios of the number of approvals and “okays” to the total number of evaluations are denoted by 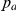 and 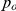, respectively. When a given user 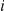 evaluates item 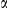 with random attributes, the average opinion is 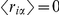 and hence without recommendation, 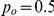. Values of 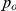 exceeding 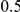 represent a working recommender system. As shown in Fig. 4, both 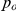 and 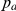 increase quickly in the early stage of the system's evolution and saturate at values considerably higher than the initial ones.

**Figure 5:**
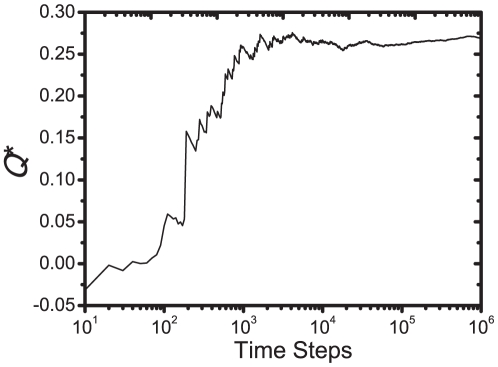
Good news live longer while bad news die out soon. Time evolution of the effective quality 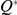 of the evaluated items. Parameter values are the same as in Fig. 3.

We next check if the average quality of the evaluated items is higher than it would be without recommendation. The intrinsic quality of item 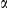 is defined as the sum of all the elements of 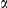
's attribute vector, 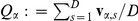; the average quality 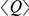 of all items is zero. We introduce the effective average quality of evaluated items, 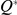, which is weighted by the number of evaluations of each item. For example, if an item with quality 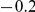 was evaluated by 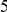 users and another item with quality 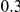 was evaluated by 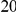 users, the corresponding value of 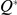 is 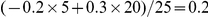. A well-performing recommender system should support spreading of high-quality items and hence 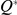 should be high. As shown in Fig. 5, 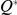 increases in our system quickly from zero to approximately 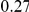. Considering that the quality value of most items is close to zero (less than 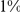 of all items have quality greater than the observed effective value 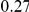), this result signifies a well-performing social filtering systems.

## Discussion

Uncovering common patterns of leader-follower networks is important for our understanding of spreading processes in social environments. We analyzed empirical data from four large-scale real social networks where the notion of leadership can be introduced and found indications of scale-free leadership structures. We studied the social recommendation model inspired by informal social recommending mechanisms (“word of mouth”) that was studied in [27]. We proposed a simplified version of this model which was shown via agent-based simulations to reproduce the observed power-law out-degree distributions. The underlying mechanism leading to these scale-free leadership structures can be summarized as “good get richer”: users with broad interests and good judgments are likely to become popular leaders for the others. In our case, broad interests are helpful to attract attention from the others while good judgments ensure reliability of the received recommendations. Although this result was obtained by a specific recommendation model, its implications go beyond social recommender systems. For example, the scale-free nature of citation networks [51], [52], [53], [54], [55], [56] might be more fundamentally explained by the present mechanism rather than by the notorious “rich get richer” mechanism [42], [43], [44]. The reason is that papers are cited by scientists not only because they have already been cited many times but mainly because they contain relevant and credible results [52]. Note that, the “rich get richer” and “good get richer” mechanisms are indeed related, depending on the criteria on goodness. For example, in evolutionary game, the criterion of a good player may be her/his cumulative wealth, and in scientific publications, the criterion of a good paper may be its cumulative citations. In such cases, the two mechanisms are not distinguishable. If only the network structure is observable, we can measure the strength of “rich get richer” mechanism [57], yet in principle we can say nothing about “good get richer” mechanism. Additional information about each node's features, attributes, fitness and functionalities may drive us to more in-depth understanding about the existence of “good get richer” mechanism. From this point of view, the “good get richer” mechanism can be considered as a deeper mechanism possibly underlying the observed “rich get richer” phenomenon in some systems.

Furthermore, our agent-based simulations reveal that the proposed model is an effective tool for quality information filtering and it is also efficient in requiring very little computation. These noticeable features are of particular relevance for resource-sharing services which are recently experiencing increasing popularity. Most of them (take *digg.com*, *reddit.com* and *wikio.com* as examples) still adopt the traditional organization in which resources are ranked by popularity and divided into categories created by a top-down approach. Known recommendation techniques are also designed in a centralized way where the systems, rather than the users, decide what to recommend to whom [58]. By contrast, systems like *delicious.com* and *twitter.com* have implemented the possibility to recommend and to have something recommended by other users. The fast growth of these online communities [59] as well as the fact that users prefer recommendations coming from their social circle [22], [23] make social recommendation a promising way to better organize and deliver online resources and to enhance online social contacts. While we neglected some relevant social factors like friendship and reciprocity and could not provide analytical solution of the proposed model, this paper offers various insights to the dynamics of resource-sharing systems and provides a starting point for their future studies.
